# Mastitomics, the integrated omics of bovine milk in an experimental model of *Streptococcus uberis* mastitis: 2. Label-free relative quantitative proteomics†
†Electronic supplementary information (ESI) available. See DOI: 10.1039/c6mb00290k



**DOI:** 10.1039/c6mb00290k

**Published:** 2016-07-14

**Authors:** Manikhandan Mudaliar, Riccardo Tassi, Funmilola C. Thomas, Tom N. McNeilly, Stefan K. Weidt, Mark McLaughlin, David Wilson, Richard Burchmore, Pawel Herzyk, P. David Eckersall, Ruth N. Zadoks

**Affiliations:** a Institute of Biodiversity , Animal Health and Comparative Medicine , College of Medical , Veterinary and Life Sciences , University of Glasgow , Jarret Building , Bearsden Road , Glasgow , G61 1QH , UK . Email: ruth.zadoks@glasgow.ac.uk; b Glasgow Polyomics, College of Medical , Veterinary and Life Sciences , University of Glasgow , Glasgow , UK; c Moredun Research Institute , Pentlands Science Park , Bush Loan , Penicuik , UK; d School of Veterinary Medicine , University of Glasgow , Glasgow , UK; e Institute of Infection , Immunity and Inflammation , University of Glasgow , Glasgow , UK; f Institute of Molecular Cell and Systems Biology , University of Glasgow , Glasgow , UK

## Abstract

Mastitis, inflammation of the mammary gland, is the most common and costly disease of dairy cattle in the western world. It is primarily caused by bacteria, with *Streptococcus uberis* as one of the most prevalent causative agents. To characterize the proteome during *Streptococcus uberis* mastitis, an experimentally induced model of intramammary infection was used. Milk whey samples obtained from 6 cows at 6 time points were processed using label-free relative quantitative proteomics. This proteomic analysis complements clinical, bacteriological and immunological studies as well as peptidomic and metabolomic analysis of the same challenge model. A total of 2552 non-redundant bovine peptides were identified, and from these, 570 bovine proteins were quantified. Hierarchical cluster analysis and principal component analysis showed clear clustering of results by stage of infection, with similarities between pre-infection and resolution stages (0 and 312 h post challenge), early infection stages (36 and 42 h post challenge) and late infection stages (57 and 81 h post challenge). Ingenuity pathway analysis identified upregulation of acute phase protein pathways over the course of infection, with dominance of different acute phase proteins at different time points based on differential expression analysis. Antimicrobial peptides, notably cathelicidins and peptidoglycan recognition protein, were upregulated at all time points post challenge and peaked at 57 h, which coincided with 10 000-fold decrease in average bacterial counts. The integration of clinical, bacteriological, immunological and quantitative proteomics and other-omic data provides a more detailed systems level view of the host response to mastitis than has been achieved previously.

## Abstract 2

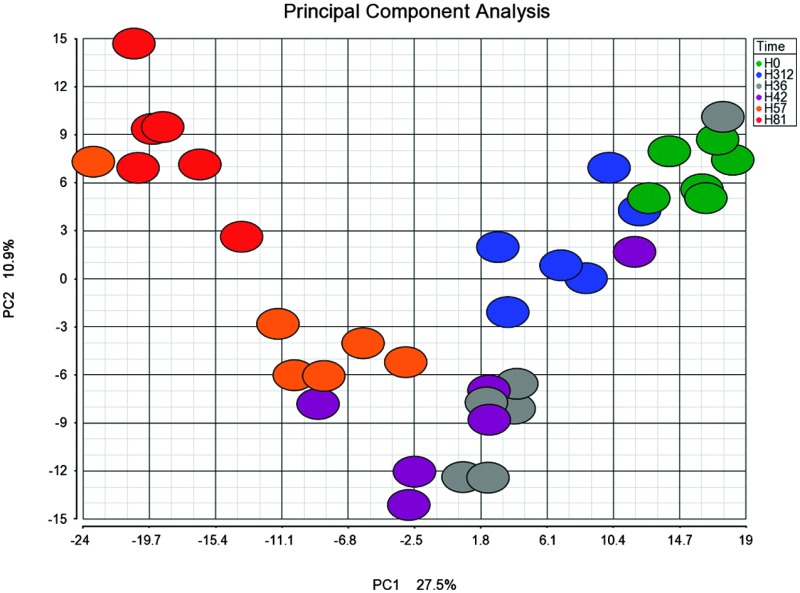
Longitudinal proteomic analysis of bovine milk shows consistent changes over time across cows after intramammary challenge with *Streptococcus uberis*.

## 1. Introduction

Bovine milk is a complex physiological secretion and contains protein at an average concentration of 32 g L^–1^. Caseins form 80% of the total milk protein while whey proteins constitute about 16% of the total milk protein.^1^ Whey comprises several hundred heterogeneous, mostly water-soluble proteins including beta-lactoglobulin, alpha-lactalbumin, blood serum albumin and immunoglobulins (IgG, IgA, IgM and IgE). These proteins have a number of functions such as ion binding, protein binding, carbohydrate binding, pattern binding, cell surface binding, lipid binding, enzyme regulating, cell-to-cell signalling and cell cycle regulating activities.^1,2^ There are substantial changes in the whey proteome (the set of proteins present in whey) during mastitis, inflammation of the mammary gland. The pathogenesis of mastitis, which is largely due to intra-mammary infections (IMI), includes an inflammatory reaction involving the release of cytokines and acute-phase proteins (APP).^3,4^ Several studies have shown changes in the milk or whey proteome due to mastitis.^5–7^

This is the second of three studies integrating omic approaches to the investigation of experimentally induced mastitis with *Streptococcus uberis*, a major cause of the disease in the UK and many other parts of the world.^8^ Using the same milk samples, temporal changes in the milk peptidome,^4^ proteome (this paper) and metabolome^9^ were determined during the acute phase of infection and its resolution. The peptidome was considered to consist of peptides, polypeptides and short protein sequences, usually degradation-derived protein fragments, with masses between ∼400 Da and ∼20 000 Da.^4^ The proteome includes whole proteins with masses ranging up to 3 MDa,^10^ which can be experimentally broken down into peptide pools using proteolytic enzymes, usually trypsin, and identified by comparing the mass spectra from experiments with the theoretical enzyme-specific fragmentation patterns derived from protein sequences. Finally, the metabolome consists of the entirety of molecules, small and large, that undergo metabolism, most of which have a mass less than 1500 Da, with the exception of lipids, which have masses up to 5000 Da.^10^

In addition to identification, quantitation of proteins in complex biological samples is possible.^10,11^ The classical method used for quantitative analysis of complex mixtures of proteins such as milk is by protein separation and comparison by two-dimensional polyacrylamide gel electrophoresis (2D-PAGE), followed by mass spectrometry (MS) analysis.^11,12^ However, the gel-based quantitative proteomics techniques are laborious and suffer poor representation of hydrophobic, very high or low molecular weight proteins.^11^ To overcome the shortcomings of the gel-based methods and to increase the dynamic range and quantitative accuracy, non-gel-based quantitative proteomics methods have been developed.^11,13^ Non-gel-based quantitative proteomics approaches can be divided into methods using metabolic or chemical labelling and label-free approaches.^13^ Some of the labelling approaches utilize isotope-labelled compounds (such as isotope labelled amino acids) that are functionally and chemically identical to the properties of their unlabelled equivalent except in mass, which allows for their discrimination in mass spectrometry. Stable labelling approaches include stable isotope labelling by amino acids in cell culture (SILAC), isotope-coded affinity tag (ICAT), isobaric tags for relative and absolute quantification (iTRAQ), dimethyl labelling and tandem mass tags.^13–15^ Label-free relative quantification is an alternative method that can be applied to clinical samples, and offers better dynamic range than some labelling approaches^15–17^ and requires minimal manipulation of samples, which reduces the possibility of introducing artefactual changes.

In this study, we describe the application of a label-free relative quantification method to analyse the quantitative changes in the proteome of bovine milk whey in the experimental model of *S. uberis* mastitis and compare those to data obtained from clinical, immunological, and peptidomic studies.

## 2. Materials and methods

Cows (*n* = 6) were challenged with *Streptococcus uberis* strain FSL Z1-048 in a single bacteriologically negative udder quarter per cow as previously described.^3^ Aliquots of milk samples collected from six time points (0, 36, 42, 57, 81 and 312 hours post-challenge (PC)) were used to generate quantitative label-free proteomics data. These were the same samples as used in the associated peptidomic^4^ and metabolomic^9^ studies and were selected on the basis of clinical manifestation, bacterial load and SCC.^3^ Body temperature of the cows and bacterial concentrations in milk from challenged quarters peaked from 24 h (bacteria) or 30 h (temperature) PC up to 57 h PC and had decreased to a plateau by 81 h PC, whereby body temperature had returned to normal and bacterial concentrations in culture positive quarters stayed constant until the end of the study at 312 h PC. The challenge study was conducted at the Moredun Research Institute (Penicuik, UK) and had received ethical approval from the Institute's Experiments and Ethical Review Committee in accordance with the UK's Animals (Scientific Procedures) Act 1986.^3^

### 2.1 Label-free quantitative proteomic data generation

#### 2.1.1 Separation of whey

The aliquots of frozen skimmed milk samples,^3^ ranging between 0.5 mL and 1.5 mL in volume per sample, were thawed at 4 °C. The volume of every sample was brought to 1.5 mL using phosphate buffered saline (PBS). To remove the residual milk fat globules and cell pellets, the samples were centrifuged at 13 000 × *g* for 30 min at 4 °C in an Eppendorf centrifuge (model 5804R) with a fixed-angle rotor (FA-45-30-11). Using a pipette, the middle clear portion (1 mL) was carefully drawn from each sample and transferred into an ultracentrifuge tube (Beckman Coulter Thickwall polycarbonate, part no. 343778) and centrifuged in a Beckman Coulter benchtop ultracentrifuge (model TL-100) with a fixed-angle rotor (TLA-100.2) at 150 000 × *g* (59 000 rpm) for 60 minutes at 4 °C. Most of the caseins in the samples sedimented to the bottom of the ultracentrifuge tubes, and above them exosomes formed a loose pellet layer with crude whey forming the supernatant. This crude whey was transferred to a clean ultracentrifuge tube and centrifuged again at 150 000 × *g* for 60 minutes at 4 °C to remove the residual caseins.^7^

#### 2.1.2 Whey protein extraction, purification and normalization

Total protein quantity in the whey was measured by Bradford protein assay in 250 μL microplate assay format using Bio-Rad protein assay dye reagent concentrate (product no. 500-0006) and bovine serum albumin (BSA) fraction V (Roche, product no. 10735086001) as the standard. To remove substances that might interfere with downstream proteomic analysis, proteins from whey were purified by precipitating them with absolute acetone.^7,18^ Using the measured total protein concentration in each sample, whey was diluted with HPLC grade water to 2 mg mL^–1^ total protein. For every diluted whey sample, an aliquot of 100 μL was transferred into a 1.5 mL micro test tube and six volumes (600 μL) of ice-cold 100% acetone (VWR International, product no. 20066.330) was added and kept at –80 °C for 12 hours. Precipitated proteins were centrifuged at 20 000 × *g* for 40 minutes at –4 °C in an Eppendorf centrifuge (model 5804R). The supernatant was discarded, and the pellets (precipitated proteins) were washed three times with 400 μL of 80% (v/v) acetone to remove salts, and then dried under a fume hood for 10 minutes. The dried pelleted proteins from each sample were re-suspended in 50 μL of 50 mM ammonium bicarbonate (Sigma-Aldrich, product no. A6141) buffer (NH_4_CO_3_ buffer) and the extracted protein quantity was measured by Bradford protein assay as described before. The re-suspended proteins in each sample were normalized by diluting them with the required volume of NH_4_CO_3_ buffer to arrive at 2.5 mg mL^–1^ total protein concentration.

#### 2.1.3 Preparation of trypsin digests

For every sample, an aliquot of 40 μL of the normalized re-suspended proteins, containing 100 μg of total proteins in buffer was transferred into a 1.5 mL micro test tube. For each aliquot, 12 μL of 10% (w/v) sodium deoxycholate (SDC) solution in buffer (Sigma-Aldrich, product no. D6750), 8 μL of 80% (v/v) acetonitrile (Fisher Scientific, product no. 10660131) in buffer and 50 μL of 10% (w/v) modified trypsin (Promega, product no. V5111) in trypsin re-suspension buffer were added. The digest was incubated for 18 hours at 37 °C in a heating block. Then, 12 μL of 1% (v/v) formic acid (Sigma-Aldrich, product no. 94318) was added to the digest (final formic acid concentration 0.1%) to precipitate SDC, and samples were centrifuged at 16 000 × *g* for 10 minutes at 4 °C. For every sample, supernatant containing 2 μg (calculated) of digested protein was transferred into a well of a conical bottom microplate and dried in a SpeedVac (Thermo Fisher Scientific, model no. SPD1010).

#### 2.1.4 On-line liquid chromatography and tandem mass spectrometry

For on-line reversed-phase liquid chromatography and mass spectrometry (LC-MS), a Dionex UltiMate 3000 RSLCnano (liquid chromatography) system coupled to a Thermo Scientific Orbitrap Elite mass spectrometer was used. A stainless steel Nano-Trap column with 300 μm inside diameter, 5 mm length, particle size 5 μm and pore size 10 nm, packed with stationary phase Acclaim PepMap C18 (Thermo Scientific, part no. 160454) and a resolving Nano LC column with 75 μm inside diameter, 15 cm length, particle size 2 μm and pore diameter 10 nm with stationary phase Acclaim PepMap RSLC C18 (Thermo Fisher Scientific, part no. 164534) were used in the HPLC. The dried protein digests in the microplate were loaded on the Rapid Separation LC (RSLC) Autosampler connected to the C18 trap column equilibrated in 96% solution A (0.1% formic acid in HPLC grade water (v/v)) and 4% solution B (80% acetonitrile and 0.08% formic acid in HPLC grade water (v/v)) with a flow rate of 25 μL min^–1^. The trap column was washed for 12 minutes at the same flow rate and then switched to the in-line resolving C18 column. A constant flow rate of 300 nL min^–1^ was maintained with a linear gradient from 4% solution B to 40% solution B in 108 minutes, then to 100% solution B by the 124th minute. Then the column was washed with 100% solution B for 5 minutes followed by recalibration with 96% solution A for 6 minutes. In the mass spectrometer, one scan cycle comprised MS1 scan (*m*/*z* range from 400–2000) in the Orbitrap Elite followed by up to 20 data-dependent MS2 scans (threshold value 1000 and maximum injection time 200 ms) in the Velos LTQ in collision-induced dissociation (CID) mode. To account for any retention time drift, carryover or other errors that might occur during the run, the sample loading order was randomized using Microsoft Excel. After every six samples, a blank was analysed to monitor carryover. All samples were run consecutively without breaks, which took about 4 days of mass spectrometer time.

### 2.2 Label-free quantitative proteomic data analysis

#### 2.2.1 Exploration of the raw data

The raw MS/MS data obtained from each sample were visually examined by generating a number of plots using MZmine (version 2.10) software.^19^ To examine sample loading and peak resolution, total ion current (TIC) chromatograms and base peak chromatograms were generated from the MS1 data obtained from each sample. To detect chromatographic shifts in retention time, MS1 spectra were visualized by generating 2D and 3D plots using the 2D and 3D plot functions in MZmine software. In addition, 2D plots and TIC chromatograms of the MS1 spectra were generated using the integrated viewer in the MaxQuant software (version 1.5.2.8)^20^ and examined for overall consistency and retention time shifts between the samples.

#### 2.2.2 Peptide identification and protein quantification

After initial quality control, the raw MS/MS data from all samples and blanks were imported into MaxQuant software (version 1.5.2.8) for label-free relative quantification analysis.^21^ Feature detection and mass recalibration were automatically performed in MaxQuant, and peptides were identified using its integrated Andromeda search engine.^22^ Reporter quantification, retention time alignment, protein assembly, label-free quantification and MaxLFQ normalization were also performed in MaxQuant.^23^ For identification and quantification, N-terminal acetylation, oxidation of methionine and deamidation of asparagine or glutamine were set as variable modifications, and carbamidomethylation of cysteines was set as a fixed modification. For *in silico* digestion, Trypsin/P was used and a maximum of 2 missed cleavages were allowed. Up to 6 ppm peptide mass tolerance was allowed during the main search. A false discovery rate (FDR) up to 1% was allowed for peptide spectrum match and protein assembly, and the FDR was estimated using the reversed peptide sequences. At least one unique or “razor” peptide was required for identification. For label-free quantification, the ‘Fast LFQ’ option was turned off and a minimum of one quantified peptide pair was required for pair-wise comparisons of a protein between two samples. The ‘match-between-runs’ option with a match time window of 2 minutes was used to transfer identifications across the replicate experiments, whereby the 6 individual cows were treated as biological replicates for each time point. In addition, absolute protein quantitation was performed using the intensity based absolute quantification (iBAQ) method.

Proteins from the *Bos taurus* proteome were identified using the 23 868 protein reference proteome (UniProt Proteome ID: UP000009136; last modified 10 May 2015), which was downloaded from the UniProt Knowledgebase and imported into the Andromeda search engine. Conflicts of multiple protein assignments were manually resolved taking into account the peptide counts, the razor and/or unique peptide counts, and the evidence status of the protein annotation (annotation score) in the UniProt database. Where a protein was identified based on comparison with both the *Bos taurus* reference proteome and the MaxQuant contaminant list, they were assigned to *Bos Taurus*, because many proteins on this list, *e.g.* keratin or bovine serum proteins, are of bovine.^24^

#### 2.2.3 Statistical analysis

Statistical analysis was performed using Perseus (version 1.5.2.6), the Partek Genomics Suite (version 6.6, Partek Inc., St. Louis) and R (version 3.1.2) software. The normalized protein intensities from the MaxQuant analysis were imported into Perseus software. Protein intensities (abundances) in the linear scale were transformed into logarithmic scale with base two. The missing values were replaced with a constant value of 10 to simulate signals from low abundant proteins. For exploratory analysis, histograms were generated to examine the dataset. Hierarchical clustering analysis and principal component analysis (PCA) were performed using Perseus and Partek Genomics Suite software. To identify differentially expressed proteins one-way analysis of variance (ANOVA) was performed with time as factor. From the ANOVA results, protein lists were created by comparing each time-point PC to the pre-challenge results (0 h PC). Proteins with an absolute fold change >2 and FDR-adjusted *p*-value < 0.05 were considered differentially expressed and included in the protein lists.

#### 2.2.4 Pathway analysis

The differentially expressed proteins were analysed for enrichment of canonical pathways using ingenuity pathway analysis (IPA) software (QIAGEN, Redwood City, CA) and the STRING database (version 10.0) and search tool.^25^ IPA computes an enrichment score for the overlap between the observed and the predicted regulated gene sets using a Fisher's exact test and *p*-value >0.05. The direction of regulation, *i.e.* up- or downregulation, was inferred from the activation *z*-score in the IPA.^26^ In addition, the ratio of identified (*i.e.* present in the sample) to potentially identifiable proteins (*i.e.* present in the pathway) is calculated for each pathway.

## 3. Results

### 3.1 Quantification and analysis of bovine proteins

TIC chromatograms, base peak chromatograms and 2D plots were generated for each sample and showed overall consistency with a retention time drift of about 2 minutes, demonstrating high quality of the raw data (Fig. S1, ESI†). A total of 2552 non-redundant bovine peptides were quantified, and 570 proteins were assembled from the quantified peptides (Table S1, ESI†). Exploratory data analysis such as histograms, hierarchical clustering analysis (HCA) and principal components analysis (PCA) were performed on the quantified protein data.

#### 3.1.1 Hierarchical clustering analysis

**Fig. 1:**
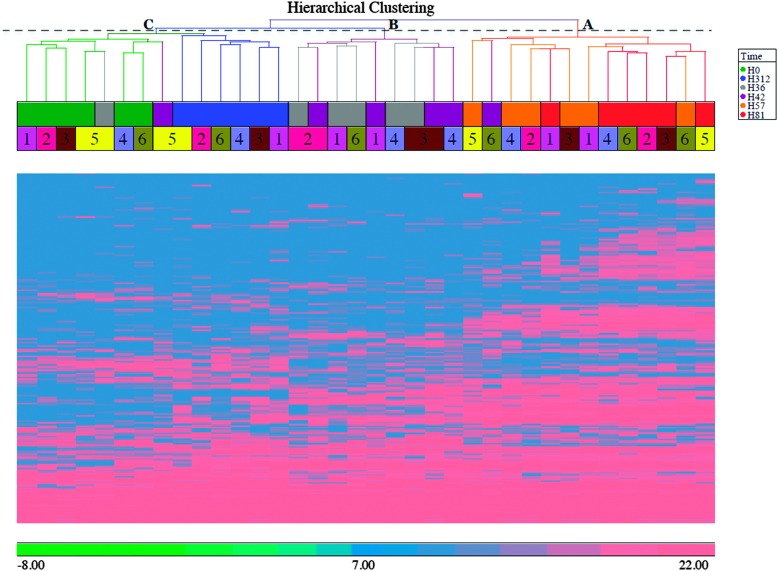
Heat map of the bovine proteome after intramammary challenge with *Streptococcus uberis* with hierarchical clustering analysis of cow and time. This heat map is based on 570 proteins. Hierarchical clustering analysis was performed using Euclidean distance as distance metric and average linkage as agglomeration method. Clusters are identified by letters (C = pre-challenge and resolution stage; B = early to peak infection based on bacterial numbers; A = post peak infection), time points by colours (see inset), and individual cows by numbers. Scale bar indicates intensity of upregulation on a log 2 scale.

To explore the dataset, HCA using Euclidean distance and average linkage was performed on the 570 proteins that were quantified from the cow reference proteome. The analysis (Fig. 1) shows three major clusters in the column dendrogram, corresponding to different phases of the infection process. Cluster C includes samples from before challenge (0 h PC) and at late resolution stage (312 h PC), by which time 5 of 6 cows had cleared the infection.^3^ It also includes 36 h and 42 h PC samples from cow 5, which was previously identified as a late responder based on clinical signs and cytokine profiling.^3^ Cluster B includes samples from 36 and 42 h PC, corresponding to the early stage of infection, which is characterized by bacterial growth and neutrophil influx.^3^ Cluster A predominantly contains samples from 57 h and 81 h PC, during which time bacterial numbers had started to decrease.^3^

#### 3.1.2 Principal component analysis

**Fig. 2:**
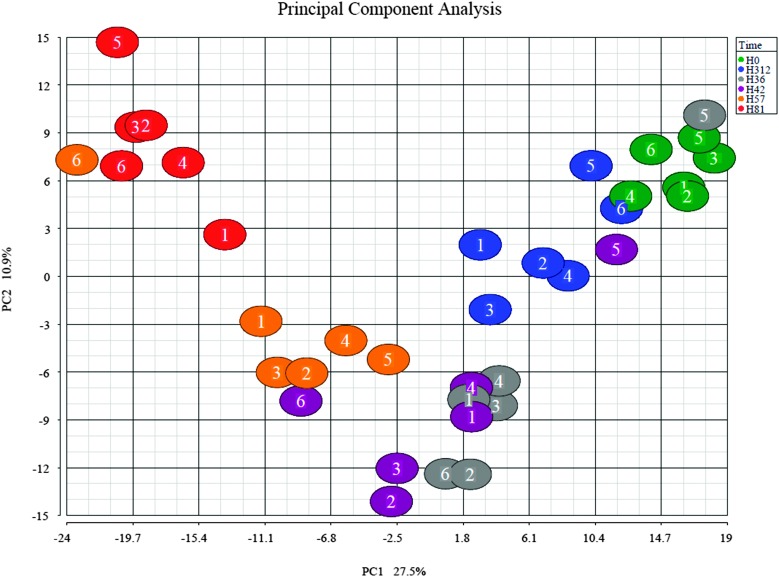
Principal component analysis (PCA) of the bovine proteome after intramammary challenge with *Streptococcus uberis*. The PCA was based on 570 proteins and the plot was generated using the Partek Genomic suite. The data points refer to milk samples obtained from 6 cows at 6 time points post challenge (PC). Cows are identified by number and time points by colour, with hours PC shown in the legend.

To further examine the set of 570 bovine proteins, PCA was performed (Fig. 2). The PCA shows clustering of samples by time-point with a few exceptions. As in HCA, results are similar for the pre-challenge (0 h) and resolution time points (312 h). Samples collected at 81 h PC were most divergent. Outliers at 36 and 42 h PC, which cluster with samples from 0 h, correspond to the slow responder (cow 5) that is also visible in Fig. 1 and in clinical, bacteriologic and inflammatory parameters.^3^

#### 3.1.3 Differential expression analysis

One-way ANOVA was performed with time as factor to identify proteins that were differentially expressed between pre- and post-challenge time points. No distinction was made between proteins that were detected in all samples and those that were detected in a subset of samples only, but this information is reflected in the LFQ intensities (quantities) listed in ESI,† Table S1 where intensity is shown as 0 if a protein is not detected. Differentially expressed protein lists were created for each time point, and proteins with an absolute fold change more than 2 and FDR-adjusted *p*-value less than 0.05 were included in the protein lists. For time points 36, 42, 57, 81 and 312 h PC, there were 76 (54 upregulated, 22 downregulated), 126 (96 upregulated, 30 downregulated), 237 (186 upregulated, 51 downregulated), 292 (248 upregulated, 44 downregulated) and 56 (49 upregulated, 7 downregulated) differentially expressed proteins, respectively (Table S2, ESI†). The top-15 upregulated and downregulated bovine proteins for each time point, as compared to 0 h PC, are given in Tables 1–5. Patterns of up- and down regulation differed both qualitatively (proteins) and quantitatively (fold change) between time points, with strongest up- and down-regulation observed at 57 and 81 h PC. Upregulated proteins include APP, *e.g.* haptoglobin and serum amyloid A (SAA); antimicrobial proteins, *e.g.* the cathelicidin family and peptidoglycan recognition protein; and proteins with dual APP and antimicrobial function, *e.g.* histidine-rich glycoprotein (HRG) and lipopolysaccharide-binding protein (LBP). Down-regulated proteins included cystatin-B, dystroglycan, and mucin-1 in the early stage of infection (36 and 42 h PC; Tables 1 and 2), and myozenin-1 and alpha-lactalbumin at the subsequent stage (57 and 81 h PC; Tables 3 and 4). During the resolution phase (312 h PC), both the number of differentially expressed proteins and the fold change were smaller than at earlier infection stages, with only 7 proteins still significantly downregulated (Table 5), and in agreement with results from HCA and PCA, which also showed a return to normal at 312 h PC.

**Table 1:** Top-15 most up-and down-regulated bovine proteins at 36 hours after intramammary challenge with *Streptococcus uberis*

Up/down-regulated	UniProt ID	Protein name	Fold change	*P*-Value^*a*^
Up	Q8SPP7	Peptidoglycan recognition protein 1	3305	4.5 × 10^–10^
Up	P54229	Cathelicidin-5	1444	1.9 × 10^–8^
Up	P56425	Cathelicidin-7	1217	1.6 × 10^–6^
Up	P22226	Cathelicidin-1	1026	2.8 × 10^–8^
Up	Q2TBU0	Haptoglobin	997	3.8 × 10^–8^
Up	F1N465	Uncharacterized protein GN = KBTBD8	527	1.5 × 10^–3^
Up	E1BCU6	Uncharacterized protein GN = TCN1	401	1.5 × 10^–6^
Up	Q9TU03	Rho GDP-dissociation inhibitor 2	313	1.6 × 10^–4^
Up	P52176	Matrix metalloproteinase-9	219	1.1 × 10^–4^
Up	P33046	Cathelicidin-4	208	2.7 × 10^–4^
Up	Q0VCG9	Pentraxin-related protein PTX3	194	1.5 × 10^–8^
Up	Q58CQ9	Pantetheinase	189	8.5 × 10^–4^
Up	G3MXK8	Uncharacterized protein (Fragment) GN = PRTN3	167	1.2 × 10^–3^
Up	Q28085	Complement factor H	134	1.6 × 10^–3^
Up	Q3SZV7	Hemopexin	131	4.9 × 10^–6^
Down	P81265	Polymeric immunoglobulin receptor	–6	2.0 × 10^–4^
Down	Q3MHX6	Protein OS-9	–6	4.9 × 10^–3^
Down	P10790	Fatty acid-binding protein, heart	–7	3.2 × 10^–4^
Down	Q8WML4	Mucin-1	–38	2.7 × 10^–3^
Down	P13696	Phosphatidylethanolamine-binding protein 1	–39	1.8 × 10^–3^
Down	Q9XSG3	Isocitrate dehydrogenase [NADP] cytoplasmic	–50	5.0 × 10^–5^
Down	Q9TUM6	Perilipin-2	–61	2.0 × 10^–3^
Down	E1BLC6	Uncharacterized protein GN = TTC17	–67	4.3 × 10^–3^
Down	F1N1D2	Uncharacterized protein GN = DMC1	–77	4.6 × 10^–3^
Down	O18738	Dystroglycan	–77	1.2 × 10^–3^
Down	P26201	Platelet glycoprotein 4	–87	1.0 × 10^–4^
Down	E1B9W6	Uncharacterized protein GN = ADCY10	–145	2.5 × 10^–3^
Down	F6PZ29	Uncharacterized protein GN = MCFD2	–191	3.1 × 10^–3^
Down	F6QEL0	Cystatin-B	–204	1.8 × 10^–4^
Down	E1BN90	Uncharacterized protein GN = ZKSCAN2	–214	4.6 × 10^–3^

^*a*^False discovery rate adjusted.

**Table 2:** Top-15 most up- or down-regulated bovine proteins at 42 hours after intramammary challenge with *Streptococcus uberis*

Up/down-regulated	UniProt ID	Protein name	Fold change	*P*-value^*a*^
Up	P54229	Cathelicidin-5	9209	1.5 × 10^–10^
Up	P56425	Cathelicidin-7	8922	1.7 × 10^–8^
Up	Q8SPP7	Peptidoglycan recognition protein 1	8453	3.7 × 10^–11^
Up	Q2TBU0	Haptoglobin	4794	5.2 × 10^–10^
Up	P22226	Cathelicidin-1	3812	7.6 × 10^–10^
Up	P33046	Cathelicidin-4	2619	1.1 × 10^–6^
Up	E1BCU6	Uncharacterized protein GN = TCN1	1292	6.1 × 10^–8^
Up	P19660	Cathelicidin-2	1159	3.9 × 10^–5^
Up	F1MCC8	Uncharacterized protein GN = NWD1	1144	5.3 × 10^–4^
Up	Q0VCG9	Pentraxin-related protein PTX3	963	4.7 × 10^–11^
Up	F1N465	Uncharacterized protein GN = KBTBD8	961	6.0 × 10^–4^
Up	F1MKS5	Histidine-rich glycoprotein	775	6.3 × 10^–6^
Up	P52176	Matrix metalloproteinase-9	708	7.1 × 10^–6^
Up	F1N1F8	Uncharacterized protein GN = CENPF	661	5.7 × 10^–3^
Up	Q9TU03	Rho GDP-dissociation inhibitor 2	614	3.8 × 10^–5^
Down	P80457	Xanthine dehydrogenase/oxidase	–15	1.1 × 10^–2^
Down	P02702	Folate receptor alpha	–35	5.6 × 10^–3^
Down	P29392	Spermadhesin-1	–42	8.1 × 10^–3^
Down	Q8WML4	Mucin-1	–44	1.8 × 10^–3^
Down	P08037	Beta-1,4-galactosyltransferase 1	–51	1.9 × 10^–3^
Down	F1MNS0	Uncharacterized protein GN = HERC1	–58	2.6 × 10^–3^
Down	P63048	Ubiquitin-60S ribosomal protein L40	–70	3.2 × 10^–3^
Down	Q0VCX2	78 kDa glucose-regulated protein	–73	2.1 × 10^–3^
Down	F1N1D2	Uncharacterized protein GN = DMC1	–77	4.6 × 10^–3^
Down	O18738	Dystroglycan	–78	1.2 × 10^–3^
Down	P13696	Phosphatidylethanolamine-binding protein 1	–87	2.3 × 10^–4^
Down	P26201	Platelet glycoprotein 4	–87	1.0 × 10^–4^
Down	F6QEL0	Cystatin-B	–97	9.3 × 10^–4^
Down	F6PZ29	Uncharacterized protein GN = MCFD2	–201	2.8 × 10^–3^
Down	E1BN90	Uncharacterized protein GN = ZKSCAN2	–230	4.1 × 10^–3^

^*a*^False discovery rate adjusted.

**Table 3:** Top-15 most up- or down-regulated bovine proteins at 57 hours after intramammary challenge with *Streptococcus uberis*

Up/down-regulated	UniProt ID	Protein name	Fold change	*P*-value^*a*^
Up	Q8SPP7	Peptidoglycan recognition protein 1	27 479	2.0 × 10^–12^
Up	P54229	Cathelicidin-5	16 618	3.4 × 10^–11^
Up	Q2TBU0	Haptoglobin	14 937	3.0 × 10^–11^
Up	P56425	Cathelicidin-7	11 877	9.1 × 10^–9^
Up	P22226	Cathelicidin-1	7281	1.4 × 10^–10^
Up	P33046	Cathelicidin-4	4753	3.0 × 10^–7^
Up	Q9TU03	Rho GDP-dissociation inhibitor 2	4748	5.0 × 10^–7^
Up	F1N1F8	Uncharacterized protein GN = CENPF	4312	5.9 × 10^–4^
Up	F1MYX5	Uncharacterized protein GN = LCP1	2578	3.9 × 10^–7^
Up	Q3ZCJ8	Dipeptidyl peptidase 1	2530	7.0 × 10^–6^
Up	P02584	Profilin-1	2404	1.0 × 10^–6^
Up	P48616	Vimentin	2155	8.2 × 10^–11^
Up	P19660	Cathelicidin-2	2104	1.2 × 10^–5^
Up	E1BI67	Uncharacterized protein GN = IL18BP	2095	9.9 × 10^–7^
Up	A5PJH7	LOC788112 protein GN = LOC788112	1967	1.9 × 10^–7^
Down	P80457	Xanthine dehydrogenase/oxidase	–172	1.4 × 10^–5^
Down	P79345	Epididymal secretory protein E1	–215	4.8 × 10^–3^
Down	O18738	Dystroglycan	–222	1.1 × 10^–4^
Down	Q32KV6	Nucleotide exchange factor SIL1	–294	8.8 × 10^–4^
Down	P29392	Spermadhesin-1	–327	1.3 × 10^–4^
Down	E1BGZ9	PHD finger protein 20-like protein 1	–337	2.8 × 10^–3^
Down	P41541	General vesicular transport factor p115	–472	1.2 × 10^–3^
Down	E1BN90	Uncharacterized protein GN = ZKSCAN2	–585	1.0 × 10^–3^
Down	F6PZ29	Uncharacterized protein GN = MCFD2	–675	3.9 × 10^–4^
Down	Q58DJ3	KIAA1984	–824	2.1 × 10^–3^
Down	P00711	Alpha-lactalbumin	–1022	4.7 × 10^–6^
Down	F1MV51	Uncharacterized protein GN = APC	–1217	1.0 × 10^–3^
Down	Q8SQ24	Myozenin-1	–3030	7.2 × 10^–4^
Down	E1BNS8	Uncharacterized protein GN = SIK1	–4741	3.0 × 10^–3^
Down	Q3ZC66	Cysteine-rich PDZ-binding protein	–6094	1.5 × 10^–3^

^*a*^False discovery rate adjusted.

**Table 4:** Top-15 most up-and down-regulated bovine proteins at 81 hours after intramammary challenge with *Streptococcus uberis*

Up/down-regulated	UniProt ID	Protein name	Fold change	*P*-value^*a*^
Up	Q2TBU0	Haptoglobin	28 858	6.1 × 10^–12^
Up	Q8SPP7	Peptidoglycan recognition protein 1	17 090	6.3 × 10^–12^
Up	P54229	Cathelicidin-5	11 722	8.0 × 10^–11^
Up	Q9TU03	Rho GDP-dissociation inhibitor 2	7794	1.8 × 10^–7^
Up	P48616	Vimentin	7549	2.2 × 10^–12^
Up	P56425	Cathelicidin-7	7316	2.6 × 10^–8^
Up	F1MYX5	Uncharacterized protein GN = LCP1	5417	7.3 × 10^–8^
Up	A6QLL8	Fructose-bisphosphate aldolase GN = ALDOA	4918	8.9 × 10^–10^
Up	E1BLI9	Protein S100-A9	4847	7.6 × 10^–13^
Up	P22226	Cathelicidin-1	4743	4.3 × 10^–10^
Up	Q5E9F7	Cofilin-1	4636	8.6 × 10^–8^
Up	Q9XSJ4	Alpha-enolase	4619	3.9 × 10^–11^
Up	Q3ZBD7	Glucose-6-phosphate isomerase	4533	5.7 × 10^–8^
Up	Q3ZCJ8	Dipeptidyl peptidase 1	3839	3.1 × 10^–6^
Up	P02584	Profilin-1	3799	3.7 × 10^–7^
Down	Q8WML4	Mucin-1	–102	2.3 × 10^–4^
Down	F1MIR2	Uncharacterized protein GN = EXOC6B	–119	7.5 × 10^–4^
Down	A8YXY3	15 kDa selenoprotein GN = SEP15	–123	1.4 × 10^–3^
Down	Q9TUM6	Perilipin-2 GN = PLIN2	–166	2.2 × 10^–4^
Down	E1BN90	Uncharacterized protein GN = ZKSCAN2	–221	4.3 × 10^–3^
Down	P29392	Spermadhesin-1	–327	1.3 × 10^–4^
Down	E1BGZ9	PHD finger protein 20-like protein 1	–337	2.8 × 10^–3^
Down	F1MMF2	Uncharacterized protein (Fragment)	–359	4.1 × 10^–3^
Down	Q3ZC66	Cysteine-rich PDZ-binding protein	–475	1.9 × 10^–2^
Down	F6PZ29	Uncharacterized protein GN = MCFD2	–799	2.9 × 10^–4^
Down	Q58DJ3	KIAA1984 OS = *Bos taurus*	–824	2.1 × 10^–3^
Down	E1B9W6	Uncharacterized protein GN = ADCY10	–2764	1.2 × 10^–5^
Down	Q8SQ24	Myozenin-1	–3030	7.2 × 10^–4^
Down	F1MV51	Uncharacterized protein GN = APC	–3282	2.5 × 10^–4^
Down	P00711	Alpha-lactalbumin	–7360	5.8 × 10^–8^

^*a*^False discovery rate adjusted.

**Table 5:** Differentially expressed proteins at 312 hours after intramammary challenge with *Streptococcus uberis*

Up/down-regulated	UniProt ID	Protein name	Fold change	*P*-value^*a*^
Up	Q2TBU0	Haptoglobin	4191	7.4 × 10^–10^
Up	G3MZ19	HRPE773-like	1254	2.6 × 10^–6^
Up	P48616	Vimentin	672	3.1 × 10^–9^
Up	P30922	Chitinase-3-like protein 1	444	2.3 × 10^–7^
Up	E1BKS1	Syndecan	403	8.7 × 10^–6^
Up	P54229	Cathelicidin-5	387	7.8 × 10^–7^
Up	F1N1Z8	Uncharacterized protein (Fragment)	348	2.6 × 10^–5^
Up	Q8SPP7	Peptidoglycan recognition protein 1	291	5.5 × 10^–7^
Up	F1MYX5	Uncharacterized protein GN = LCP1	246	8.7 × 10^–5^
Up	P22226	Cathelicidin-1	226	2.4 × 10^–6^
Up	Q8SQ28	Serum amyloid A protein	220	2.6 × 10^–6^
Up	Q2HJF0	Similar to Serotransferrin	210	3.1 × 10^–5^
Up	Q9XSJ4	Alpha-enolase	190	6.7 × 10^–7^
Up	G3X746	Uncharacterized protein (Fragment) GN = CABIN1	183	4.6 × 10^–3^
Up	P33046	Cathelicidin-4	175	3.9 × 10^–4^
Down	E1BAU6	Uncharacterized protein GN = INPP5E	–2	2.1 × 10^–3^
Down	P02192	Myoglobin	–2	6.3 × 10^–4^
Down	P80195	Glycosylation-dependent cell adhesion molecule 1	–3	3.8 × 10^–3^
Down	Q0IIH5	Nucleobindin 2	–4	3.9 × 10^–5^
Down	E1BLC6	Uncharacterized protein GN = TTC17	–67	4.3 × 10^–3^
Down	P13696	Phosphatidylethanolamine-binding protein 1	–87	2.3 × 10^–4^
Down	Q8SQ24	Myozenin-1	–642	4.9 × 10^–3^

^*a*^False discovery rate adjusted.

#### 3.1.4 Pathway analysis

**Fig. 3:**
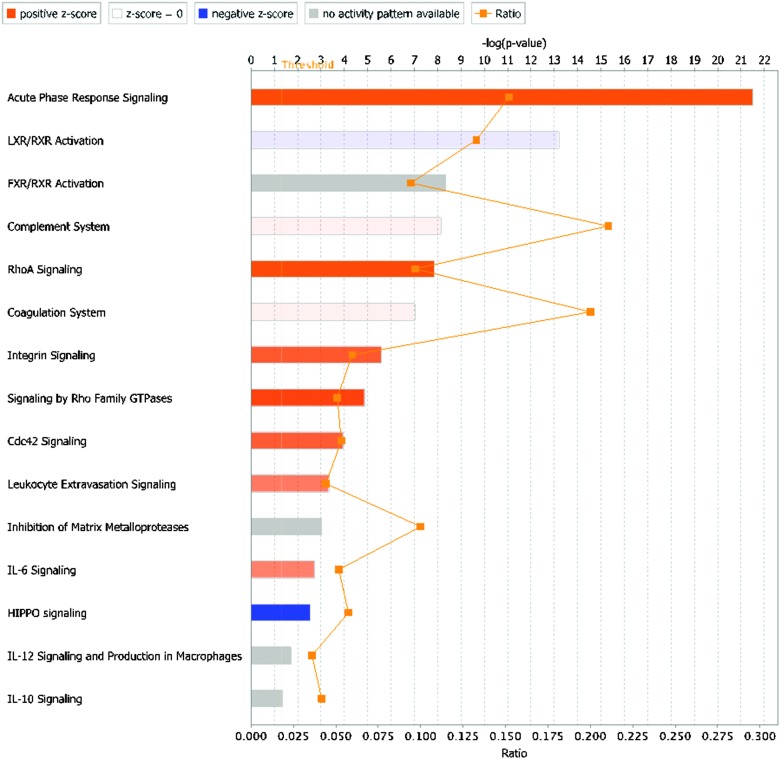
Canonical pathways enriched in the differentially expressed bovine proteins (*n* = 292) at 81 hours after intramammary challenge with *Streptococcus uberis*. The length of the bar against each pathway shows the negative log of the *p*-value obtained by a Fisher's exact test (the significance of enrichment; the longer the better), and the colour of the bar indicates the direction and strength of regulation inferred from the activation *z*-score (orange: upregulation, blue: downregulation, grey: no activity pattern available; white: *z*-score = 0, indicating upregulation of some proteins and downregulation of others), with colour intensity indicating the strength of the effect. The ratio is the proportion of proteins out of the entire pathway that were identified in the dataset, *e.g.* for ratio = 0.10, 10% of proteins from the pathway were identified in the dataset. LXR = liver X receptor, RXR = retinoid X receptor, FXR = Farnesoid X receptor, IL = interleukin.

To detect enriched canonical pathways and to construct functional networks in the differentially expressed bovine proteins, IPA was used (Fig. 3 and ESI,† Fig. S2–S5). The acute-phase response signalling pathway was the most enriched pathway at each time point, with a positive *z*-score indicating upregulation. The liver X receptor (LXR), retinoid X receptor (LXR) and Farnesoid X receptor (FXR) activation pathways were also enriched following intramammary challenge. The complement system pathway showed a change from downregulation at 36 h PC to upregulation at 81 h PC. Interleukin (IL) 6 signalling is upregulated at 57 and 81 h PC only. Other pathways are also up-regulated at those time points, including Rho signalling, integrin signalling and leucocyte extravasation signalling, whilst an additional pathway is up-regulated at 81 h PC only, *i.e.* Cdc42 signalling (Fig. 3).

The expression of 38 proteins in the acute-phase response signalling pathway changed over the course of the infection (Table 6), with maximum upregulation observed from as early as 42 h, *e.g.* for HRG and alpha-2-macroglobulin, to as late as 312 h for complement C1 subcomponent and retinol-binding protein. Less than half of the proteins (*n* = 16) were significantly upregulated at all time points PC. Of proteins with more than 10-fold upregulation, 5 were most strongly upregulated at 42 h, 6 at 57 h, 11 at 81 h, and 2 at 312 h. Haptoglobin was the most strongly upregulated protein at all time points PC. SAA was also strongly upregulated but differences were observed between different isoforms, whereby SAA4 showed a modest peak at 42 h PC whilst SAA1 and SAA3 showed much stronger and later peaks in upregluation, *i.e.* over a 1000-fold at 81 h PC. Interleukin-1 receptor agonist was the only protein that was upregulated at 36 through 81 h PC and hard returned to the pre-challenge value during the resolution phase at 312 h. Unlike APP, the antimicrobial proteins showed strong upregulation at all time points and all reached peak expression increases of several 1000 or 10 000 fold at 57 h PC. By 312 h PC, their upregulation levels had decreased to several 100 fold or less.

**Table 6:** Temporal changes in acute phase proteins and antimicrobial proteins in the bovine whey proteome after intramammary challenge with *Streptococcus uberis*. Acute phase proteins were identified using the ingenuity pathway analysis database with fold-change (compared to 0 h post challenge, PC) and *p*-values based on one-way ANOVA (show in italics if not <0.05). Antimicrobial proteins were added for comparison. For proteins with a fold change >10, the time point with strongest up- or down regulation is in bold. Values >10 are rounded to the nearest integer

UniProt accession	Protein name	Fold change at specified time PC (h)					False discovery rate adjusted *p*-value at specified time PC (h)				
		36	42	57	81	312	36	42	57	81	312
Acute phase proteins											
Q3SZR3	Alpha-1-acid glycoprotein	1.6	1.8	1.8	1.8	1.2	*1 × 10* ^*–1*^	*6 × 10* ^*–2*^	*5 × 10* ^*–2*^	*5 × 10* ^*–2*^	*5 × 10* ^*–1*^
P28800	Alpha-2-antiplasmin	4.9	5.9	4.6	3.1	1.4	4 × 10^–5^	8 × 10^–6^	7 × 10^–5^	2 × 10^–3^	*4 × 10* ^*–1*^
P12763	Alpha-2-HS-glycoprotein	1.4	1.8	1.7	1.2	–1.4	*6 × 10* ^*–2*^	3 × 10^–3^	6 × 10^–3^	*4 × 10* ^*–1*^	*6 × 10* ^*–2*^
Q7SIH1	Alpha-2-macroglobulin	68	**170**	128	102	33	2 × 10^–4^	2 × 10^–5^	4 × 10^–5^	7 × 10^–5^	2 × 10^–3^
P15497	Apolipoprotein A-I	6.3	8.0	6.8	4.1	1.5	3 × 10^–5^	5 × 10^–6^	2 × 10^–5^	7 × 10^–4^	*3 × 10* ^*–1*^
P81644	Apolipoprotein A-II	11	22	14	5.1	–1.4	4 × 10^–2^	1 × 10^–2^	3 × 10^–2^	*2 × 10* ^*–1*^	*8 × 10* ^*–1*^
Q0VCX1	Complement C1s subcomponent	1.0	1.0	2.2	20	**31**	*1 × 10* ^*+0*^	*1 × 10* ^*+0*^	*4 × 10* ^*–1*^	4 × 10^–3^	1 × 10^–3^
Q3SYW2	Complement C2	11	8.7	19	**84**	81	2 × 10^–2^	4 × 10^–2^	6 × 10^–3^	1 × 10^–4^	1 × 10^–4^
Q2UVX4	Complement C3	1.3	1.3	1.3	1.4	2.0	*1 × 10* ^*–1*^	*1 × 10* ^*–1*^	*1 × 10* ^*–1*^	*6 × 10* ^*–2*^	4 × 10^–4^
F1MY85	Complement C5a anaphylatoxin	32	32	**210**	129	21	2 × 10^–2^	2 × 10^–2^	4 × 10^–4^	1 × 10^–3^	3 × 10^–2^
P81187	Complement factor B	3.2	4.1	7.4	8.2	2.8	1 × 10^–4^	6 × 10^–6^	1 × 10^–8^	4 × 10^–9^	4 × 10^–4^
F1N076	CP Protein	3.5	4.2	4.4	3.7	2.9	3 × 10^–5^	4 × 10^–6^	3 × 10^–6^	2 × 10^–5^	3 × 10^–4^
P50448	Factor XIIa inhibitor	–2.5	–2.4	–3.0	–3.2	–1.2	6 × 10^–3^	7 × 10^–3^	1 × 10^–3^	6 × 10^–4^	*6 × 10* ^*–1*^
P02676	Fibrinogen beta chain	1.2	1.9	**13**	9.9	7.5	*8 × 10* ^*–1*^	*2 × 10* ^*–1*^	*2 × 10* ^–5^	1 × 10^–6^	5 × 10^–4^
F1MGU7	Fibrinogen gamma-B chain	–1.7	1.1	3.4	2.9	3.1	*2 × 10* ^*–1*^	*9 × 10* ^*–1*^	3 × 10^–3^	7 × 10^–3^	5 × 10^–3^
Q2TBU0	Haptoglobin	997	4794	14 937	**28 858**	4191	4 × 10^–8^	5 × 10^–10^	3 × 10^–11^	6 × 10^–12^	7 × 10^–10^
Q3SZV7	Hemopexin	131	153	**170**	158	73	5 × 10^–6^	3 × 10^–6^	2 × 10^–6^	3 × 10^–6^	3 × 10^–5^
Q3T0D0	Heterogeneous nuclear ribonucleoprotein K	1.0	4.7	2.5	**66**	1.0	*1 × 10* ^*+0*^	*1 × 10* ^*–1*^	*3 × 10* ^*–1*^	*8 × 10* ^–5^	*1 × 10* ^*+0*^
F1MKS5	Histidine-rich glycoprotein	106	**775**	760	451	30	6 × 10^–4^	6 × 10^–6^	7 × 10^–6^	2 × 10^–5^	9 × 10^–3^
F1MNW4	Inter-alpha-trypsin inhibitor heavy chain H2	51	**143**	78	52	38	3 × 10^–3^	3 × 10^–4^	1 × 10^–3^	3 × 10^–3^	5 × 10^–3^
Q3T052	Inter-alpha-trypsin inhibitor heavy chain H4	14	21	34	**38**	16	5 × 10^–3^	1 × 10^–3^	3 × 10^–4^	2 × 10^–4^	3 × 10^–3^
Q0VC51	Interleukin 1 receptor accessory	2.4	2.4	213	**267**	1.0	*3 × 10* ^*–1*^	*2 × 10* ^*–1*^	5 × 10^–8^	2 × 10^–8^	*1 × 10* ^*+0*^
O77482	Interleukin-1 receptor antagonist	30	80	**325**	176	1.0	2 × 10^–4^	8 × 10^–6^	7 × 10^–8^	5 × 10^–7^	*1 × 10* ^*+0*^
Q2TBI0	Lipopolysaccharide-binding protein	28	84	395	693	113	2 × 10^–4^	5 × 10^–6^	2 × 10^–8^	4 × 10^–9^	2 × 10^–6^
C4T8B4	Pentaxin	13	7.2	45	**82**	1.0	*6 × 10* ^*–2*^	*2 × 10* ^*–1*^	8 × 10^–3^	3 × 10^–3^	*1 × 10* ^*+0*^
P06868	Plasminogen	31	33	**76**	71	13	2 × 10^–2^	2 × 10^–2^	4 × 10^–3^	4 × 10^–3^	*7 × 10* ^*–2*^
P00978	Protein AMBP	16	5.1	**26**	16	1.2	4 × 10^–2^	*2 × 10* ^*–1*^	2 × 10^–2^	4 × 10^–2^	*9 × 10* ^*–1*^
P18902	Retinol-binding protein 4	2.3	2.2	–1.4	2.4	23	*4 × 10* ^*–1*^	*4 × 10* ^*–1*^	*7 × 10* ^*–1*^	*4 × 10* ^*–1*^	2 × 10^–3^
Q29443	Serotransferrin	4.3	5.4	5.1	4.0	2.2	2 × 10^–4^	3 × 10^–5^	5 × 10^–5^	4 × 10^–4^	3 × 10^–2^
A6QPQ2	Serpin A3-8	20	158	246	**283**	37	3 × 10^–2^	5 × 10^–4^	2 × 10^–4^	2 × 10^–4^	10 × 10^–3^
G8JKW7	SERPINA3 Protein	2.7	3.0	2.9	4.0	2.8	2 × 10^–3^	1 × 10^–3^	1 × 10^–3^	8 × 10^–5^	2 × 10^–3^
P02769	Serum albumin	1.9	2.2	2.1	1.4	–1.4	6 × 10^–3^	1 × 10^–3^	2 × 10^–3^	*2 × 10* ^*–1*^	*1 × 10* ^*–1*^
F1MMW8	Serum amyloid A protein – M-SAA3.2	20	58	107	358	73	5 × 10^–4^	1 × 10^–7^	1 × 10^–6^	1 × 10^–8^	4 × 10^–6^
P35541	Serum amyloid A protein – SAA1	5	49	1178	**1926**	6.5	*1 × 10* ^*–1*^	2 × 10^–3^	6 × 10^–7^	2 × 10^–7^	*1 × 10* ^*–1*^
Q8SQ28	Serum amyloid A protein – SAA3	93	201	556	**1585**	220	4 × 10^–5^	3 × 10^–6^	2 × 10^–7^	8 × 10^–9^	3 × 10^–6^
Q32L76	Serum amyloid A protein – SAA4	17	**66**	27	10	2.0	4 × 10^–2^	3 × 10^–3^	2 × 10^–2^	*9 × 10* ^*–2*^	*6 × 10* ^*–1*^
O46375	Transthyretin	2.4	2.2	1.9	1.3	–1.2	3 × 10^–3^	7 × 10^–3^	3 × 10^–2^	*3 × 10* ^*–1*^	*5 × 10* ^*–1*^

Antimicrobial proteins											
P22226	Cathelicidin-1	1026	3812	**7281**	4743	226	3 × 10^–8^	8 × 10^–10^	1 × 10^–10^	4 × 10^–10^	2 × 10^–6^
P19660	Cathelicidin-2	78	1159	**2104**	1683	38	6 × 10^–3^	4 × 10^–5^	1 × 10^–5^	2 × 10^–5^	2 × 10^–2^
P33046	Cathelicidin-4	208	2619	**4753**	2963	175	3 × 10^–4^	1 × 10^–6^	3 × 10^–7^	8 × 10^–7^	4 × 10^–4^
P54229	Cathelicidin-5	1444	9209	**16 618**	11 722	387	2 × 10^–8^	2 × 10^–10^	3 × 10^–11^	8 × 10^–11^	8 × 10^–7^
P56425	Cathelicidin-7	1217	8922	**11 877**	7316	178	2 × 10^–6^	2 × 10^–8^	9 × 10^–9^	3 × 10^–8^	3 × 10^–2^
Q8SPP7	Peptidoglycan recognition protein 1	3305	8453	**27 479**	17 090	291	5 × 10^–10^	4 × 10^–11^	2 × 10^–12^	6 × 10^–12^	6 × 10^–7^

## 4. Discussion

In the present study, a label-free quantitative proteomics approach was used for profiling the bovine whey proteome during experimentally induced *S. uberis* mastitis. This enabled the dynamic change in 570 proteins of the whey proteome to be studied in synchronisation with the clinical and bacteriological manifestations of infection,^3^ the peptidome^4^ and the metabolome,^9^ and allowed quantification of the relative change in multiple proteins in milk samples from the *S. uberis* infected quarters. Furthermore, by examining sequential time points following bacterial challenge, the temporal changes in important host response pathways were revealed. Thus at 36 h post challenge, the first time point examined, peptidoglycan recognition protein 1 and the cathelicidins, which are antimicrobial proteins (AMP) derived from phagocytic polymorphonuclear leucocytes (PMNL) cells that cross from the blood into the mammary gland, show the highest fold increase, reaching a peak at 57 h PC. In contrast, APP such as haptoglobin, LBP and SAA, derived from local synthesis in mammary epithelia increase at a slower rate but showed their maximal levels by 81 h PC. The concentrations in the milk samples of haptoglobin and serum amyloid A were also measured by immunoassay,^4^ giving similar increases at 57 h and 81 h and thus serving as validation of the results obtained by the quantitative proteomics approach. It would be of great interest to similarly validate by immunoassay or Western blot the results obtained for other potential biomarkers over the course of the *S. uberis* IMI, such as the cathelicidins and histidine rich glycoprotein, but this was beyond the scope of the current investigation.

Changes in the milk proteome during mastitis due to infection with *S. uberis*, *S. aureus* or *E. coli* have been studied previously using mass spectrometry techniques.^27^ Many of these studies used gel-based techniques, which are semi-quantitative, although recently quantification using labeling such as iTRAQ or calibration standards have been described.^7,28–31^ The method used here was able to yield relative quantification of 570 proteins, which is among the highest number that have been determined, being exceeded only in the study of Reinhardt and coworkers^7^ who examined subsets of milk proteins and also depleted both caseins and lactoglobins in order to enhance detection of low abundance protein. In the current study only caseins were depleted by ultracentrifugation. Method refinements introduced here that may have enhanced protein recovery included total protein concentration being normalized after acetone precipitation and the preparation of trypsin digests using SDC as well as acetonitrile to improve complete digestion of proteins.^32,33^

**Fig. 4:**
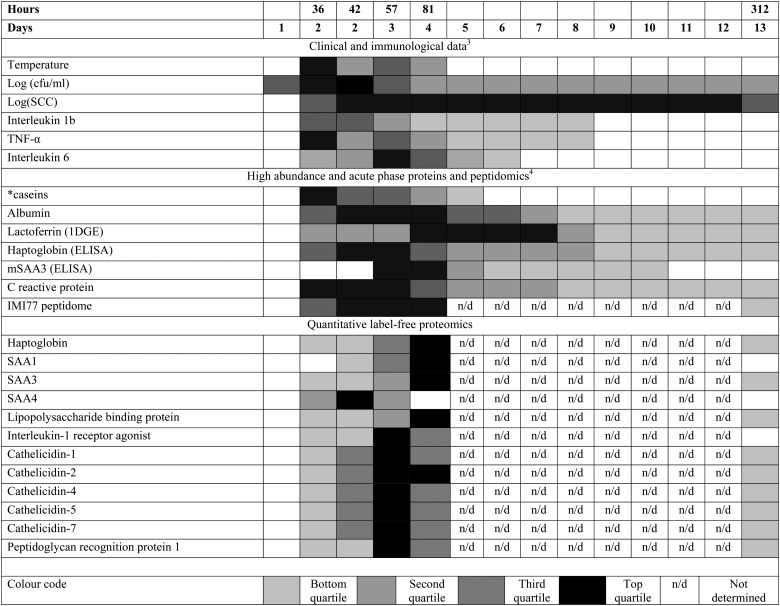
The relative responses of analytes following experimental induced *S. uberis* mastitis combining proteomic results with data from Tassi *et al.* ^3^ and Thomas *et al.* ^4^ The shading represents strength of the response relative to the peak response Responses were increases compared to day 0 levels except for casein levels (indicated by *), which decreased after challenge. cfu = colony forming unit; SCC = somatic cell count; TNF = tumor necrosis factor; 1DGE = 1 dimensional gel electrophoresis; (m)SAA3 = (milk) derived serum amyloid A; IMI77 = peptidomic profile based on 77 peptides.

In a systems biology approach it is appropriate to consider the time course of the changes in the multiple components of milk during IMI caused by *S. uberis* to put the data generated into context (Fig. 4). Many but not all of the proteome responses to IMI found by quantitative proteomics occurred at the same time as the maximal change in peptidomic and metabolomic responses^4,9^ with the maximal change in analytes occurring at 81 h PC, at 45 h after the peak in bacterial count (36 h PC) and, with the exception of one cow (cow 5), after rectal temperature had returned to normal levels. This course of events, combined with the cytokine profiles,^3^ supports the interpretation that the response to bacterial challenge first leads to cytokine release which subsequently causes the resultant change in peptide, protein and metabolite profiles. At 36 h PC, bacterial counts peak, clinical signs are detectable, cytokines IL-1, IL-6, IL-8, IL-10, and IL-12p40 have been released as well as TNF-α and there are detectable changes in the proteins and metabolites. This coincides with massive influx of polymorphonuclear leucocytes (PMNL) into the mammary gland,^3^ which accounts for the increased milk somatic cell count (SCC). The PMNL influx may be a causative event in both the reduction in bacterial numbers and the change in peptidomic and proteomic profiles.

The bioinformatics tools used here to examine the overall changes taking place in the whey proteome demonstrate that maximal responses occurred at 57 and 81 h PC, time points that clustered by HCA. PCA demonstrated that milk samples from 81 h PC were the most divergent from the pre-challenge samples while samples from 312 h PC, *i.e.* the resolution phase, were being restored towards, but were still distinct from the pre-challenge clusters, even though 5 cows had cleared the infection at that point.^3^ Pathway analysis using IPA identified the APP pathway as having the largest change of any pathway at all time points, supporting the evidence from investigations using immunoassay and transcript analysis that these proteins are among those most affected by IMI.^4,34^ The second and third most affected pathways were the LXR/RXR activation and FXR/RXR activation pathways, incorporating liver (LXR), retinoid (RXR) and farnesoid (FXR) receptor related proteins. However, a number of APP are also components of these pathways and lead to identified up-regulation by IPA due to this cross-recognition. The IPA also showed that although the PMNL influx increases rapidly between 24 and 42 h post-challenge,^3^ the leucocyte extravasation signalling pathway was only enriched at 57 and 81 h PC, indicating that there may be a lag between initial influx and detectable levels of protein upregulation in this pathway. Similarly, IL-6 levels were significantly elevated at 36 and 42 h PC based on ELISA assays,^3^ but enrichment of the IL-6 pathway was not detected until 57 h PC by proteomic analysis and IPA.

Like IPA, analysis of differential protein expression profiles identified APP as being central to the pathophysiological changes following *S. uberis* challenge. In addition, several AMP featured in the lists of proteins with the highest fold increase in expression. The AMP are a diverse group of proteins that show antimicrobial activity. They are secreted by PMNL and function as primary effectors of innate immunity in the mammary gland.^35^ Among the AMP, cathelicidins and peptidoglycan recognition protein 1 were strongly upregulated from 36 h PC onwards, with expression levels 1000s of times higher than before challenge. Indeed, cathelecidin-5 and peptidoglycan recognition protein showed the largest fold increase of any of the proteins quantified by LC-MS/MS up to and including 57 h PC. Previous studies also reported up-regulation of AMPs, particularly cathelicidins, in mastitic milk.^6,7^ Other AMPs, *e.g.* lactoperoxidase and mucin, which is thought to be an inducible innate immune effector,^36^ were detected at lower level after challenge, which could indicate decreased expression, or increased use without replenishment. Interestingly, the highest levels of cathelicidins were detected from 42 to 81 h, a period that coincides with a massive decrease in bacterial numbers^3^ from an average of 10^8^ cfu ml^–1^ down to 10^4^ cfu ml^–1^, and cathelicidin expression decreased after this reduction in cfu count. Unlike some other mastitis pathogens, *S. uberis* is resistant to phagocytosis and killing by neutrophils.^37^ The massive increase in cathelicidin levels, which followed PMNL influx and preceded or coincided with bacterial clearance, may provide an alternative mechanism by which PMNL contribute to resolution of IMI caused by *S. uberis*.

As the acute-phase response is a swift systemic inflammatory reaction in response to infections and is already implicated in responses to IMI^38,39^ it is no surprise that changes were found among the APP in this investigation. However, the profile of changes in multiple APP, in response to the *S. uberis* challenge, was shown here in much more detail than has been previously possible and within the APP, differing profiles were found. A number of the APP showed their highest fold increase at 42 h PC. Thus, alpha-2-macroglobulin and HRG had fold changes of 170× and 775× respectively at this time point. In contrast, a number of APP showed continuing elevation in their fold increase up to 81 h PC with haptoglobin, SAA1 and LBP having fold increases of 28 858×, 1926× and 693× respectively. The differences found in the profile of responses of the APP are likely to be due to cellular mechanisms in the control of their synthesis and release, dependent on the cytokine cocktail developed in response to infection.^40,41^ Cytokine profiles differ between bacterial species^42^ and hence differing profiles of both the APP and AMP responses can be expected for different mastitis pathogens. Further investigation of these profiles and of interaction with the peptide and metabolomics changes^4,9^ may lead to multiplexed biomarker analysis capable of providing pathogen specific diagnosis which would be of great value in mastitis diagnosis and therapy.

Examining the expression of individual APP, increased expression of haptoglobin is known to occur during mastitis^38,43^ and has been quantified previously in proteomic investigation.^44,45^ It was apparent that Hp detection by quantitative proteomic analysis was more sensitive than detection by ELISA, as substantial increases in Hp levels were detected at 36 h PC in by the proteomic approach, but not by Thomas and colleagues where ELISA was used.^4^ The high fold increase of Hp which was still present at 312 h PC at 4191× indicates that Hp may be useful as an indicator of high SCC, which was still high at that time, but may have limited value as indicator of the IMI, which had been resolved in 5 of 6 animals.^3^ SAA, in the isoforms found here, also reached a maximum at 81 h PC. These are a family of apolipoproteins that are associated with high density lipoprotein when in serum.^38^ The mammary associated SAA3 isoform is also one of the first APP reported to increase during mastitis and previous proteomics studies have shown up-regulation of isoforms of SAA in milk in response to gram-negative and gram-positive pathogens.^6,7,38^ As for Hp, proteomic analysis identified the increase in SAA levels earlier than ELISA-based analysis^4^ demonstrating further that quantitative proteomics may be more sensitive than the forms of ELISA used previously. However the use of relative quantitation may give a misleading impression of the change taking place when the level of the protein in the control (0 h PC) is very low or not detectable in the LC-MS/MS analysis. Absolute quantification by calibrated standard in quantitative proteomics or in immunoassay is needed to determine the change in the absolute concentration of the milk proteins in IMI.

Among the APP with an early maximum fold increase at 42 h PC, alpha-2-macroglobulin is a protease inhibitor that can inhibit all four classes of proteases (serine, cysteine, aspartyl and metalloproteases). It is present in milk in its native, active state and its concentration is known to increase during mastitis.^7,46^ HRG was also identified as an early elevated APP and is a major plasma protein in a range of mammals, including cattle.^47,48^ It plays a role in blood coagulation, fibrinolysis, and innate immune systems and is also thought to have antibacterial properties.^48^ As HRG was upregulated as early as 36 h PC and was returned to normal levels towards the end of the experiment, it may have a role as a diagnostic marker in detecting the occurrence and resolution of IMI. However, the only protein with significantly increased expression at 36 h PC which had returned to pre-challenge levels in the resolution phase was interleukin-1 receptor agonist. During the resolution phase of IMI (57 to 312 h PC), increased levels of vimentin were detected. Vimentin is a fibroblast marker, whilst there are conflicting reports on its presence in myoepithelial cells.^49,50^ Its elevated expression in milk would appear to indicate damage or repair of the subalveolar tissue of the mammary gland.

In addition to quantifying host proteins in whey, we attempted to quantify bacterial peptides and identify bacterial proteins using the *S. uberis* reference proteome (data not shown). Despite massive increase in bacterial numbers over the course of infection with peak concentrations around 10^8^ colony forming units per ml of milk,^3^ differential expression analysis showed much lower fold increases than for bovine proteins (maximum of 706 fold increase for a bacterial putative lipoprotein *versus* maximum of 28 858 fold change for haptoglobin). Separation of bacteria from whey or other modifications to the sample processing methods may be needed for better characterisation of the bacterial proteome during IMI.

## 5. Conclusion and outlook

Using a label-free relative quantification method, changes in protein expression in bovine whey in an experimental model of *S. uberis* mastitis have been determined. In particular, the dynamic changes in the proteome during establishment and resolution of infection, with emphasis on APP and AMP has been determined. Our results were in agreement with previous proteomic studies but provide a time-course rather than a snapshot of protein profiles. Proteins that have not been previously associated with mastitis, including HRG, an acute-phase and antimicrobial protein, have been quantified. In addition, the time course of events observed in our linked studies provides a potential explanation for the clearance of *S. uberis* after influx of PMNL, whereby cathelicidins produced by the PMNL rather than neutrophil phagocytosis and killing may be the main effector mechanism. Quantitative proteomics has provided an additional layer of analysis to the milk whey samples obtained during the experimental model of bovine mastitis caused by *S. uberis* and by integration with the pathophysiological, molecular, peptidomic and metabolomics analyses performed on the same sample set has enabled a more comprehensive, systems level view of the host responses to bovine mastitis than has been achieved previously.
