# Supplementary material for: Mastitomics, the integrated omics of bovine milk in an experimental model of Streptococcus uberis mastitis: 2. Label-free relative quantitative proteomics
Source: Mol Biosyst. 2016 Jul 14;12(9):2748–61. doi: 10.1039/c6mb00290k (PMC5048399; doi:10.1039/c6mb00290k)
Supplement: Supplementary file 1 [file MB-012-C6MB00290K-s001.zip › ESI_captions.docx]

**Supplementary Electronic Information Captions**

**Table S1.** Peptides and proteins identified through comparison with *Bos taurus* proteome after intramammary challenge with *Streptococcus uberis*

**Table S2.** Differentially expressed bovine proteins at 36, 42, 57, 81 or 312 hrs after intramammary challenge with *Streptococcus uberis*

**Figure S1**. TIC chromatograms showing the complexity of the samples, events occurring during the runs, peptide elution profiles and efficiency in the use of instrument time. Superimposed base peaks from all the runs show comparison between the runs. The chromatogram from each individual sample is plotted using a different colour. The inset diagram shows TIC chromatograms for milk samples from all 6 cows at 81 hrs post challenge with retention time of 52 to 54 minute. Some peaks are annotated with m/z values, and they show retention time drift of up to 2 minutes. Minor differences in m/z values (mass accuracy) can also be seen.

**Figure S2**. Canonical pathways enriched in the differentially expressed bovine proteins (n = 76) at 36 hours after intramammary challenge with *Streptococcus uberis.* The length of the bar against each pathway shows the negative log of the p-value obtained by a Fisher’s exact test (the significance of enrichment; the longer the better), and the colour of the bar indicates the direction and strength of regulation inferred from the activation *Z*-score (orange: upregulation, grey: no activity pattern available; blue: downregulated; white: z-score = 0, indicating upregulation of some proteins and downregulation of others), with intensity of colour indicating the strength of the effect. Ratio indicates the proportion of proteins out of the entire pathway that were identified in the dataset, e.g. for ratio = 0.10, 10% of proteins from the pathway were identified in the dataset. LXR = liver X receptor, RXR = retinoid X receptor, FXR = Farnesoid X receptor, LPS = lipopolysaccharide, IL = interleukin.

**Figure S3**. Canonical pathways enriched in the differentially expressed bovine proteins (n = 126) at 42 hours after intramammary challenge with *Streptococcus uberis.* The length of the bar against each pathway shows the negative log of the p-value obtained by a Fisher’s exact test (the significance of enrichment; the longer the better), and the colour of the bar indicates the direction and strength of regulation inferred from the activation *Z*-score (orange: upregulation, grey: no activity pattern available; blue: downregulated; white: z-score = 0, indicating upregulation of some proteins and downregulation of others), with intensity of colour indicating the strength of the effect. Ratio indicates the proportion of proteins out of the entire pathway that were identified in the dataset, e.g. for ratio = 0.10, 10% of proteins from the pathway were identified in the dataset. LXR = liver X receptor, RXR = retinoid X receptor, FXR = Farnesoid X receptor, LPS = lipopolysaccharide, IL = interleukin, TR = thyroid receptor.

**Figure S4**. Canonical pathways enriched in the differentially expressed bovine proteins (n = 237) at 57 hours after intramammary challenge with *Streptococcus uberis*. The length of the bar against each pathway shows the negative log of the p-value obtained by a Fisher’s exact test (the significance of enrichment; the longer the better), and the colour of the bar indicates the direction and strength of regulation inferred from the activation *Z*-score (orange: upregulation, grey: no activity pattern available; blue: downregulated; white: z-score = 0, indicating upregulation of some proteins and downregulation of others), with intensity of colour indicating the strength of the effect. Ratio indicates the proportion of proteins out of the entire pathway that were identified in the dataset, e.g. for ratio = 0.10, 10% of proteins from the pathway were identified in the dataset. LXR = liver X receptor, RXR = retinoid X receptor, FXR = Farnesoid X receptor, IL = interleukin, PPAR = peroxisome proliferator-activated receptor

**Figure S5**. Canonical pathways enriched in the differentially expressed bovine proteins (n = 56) at 312 hours after intramammary challenge with *Streptococcus uberis*. The length of the bar against each pathway shows the negative log of the p-value obtained by a Fisher’s exact test (the significance of enrichment; the longer the better), and the colour of the bar indicates the direction and strength of regulation inferred from the activation *Z*-score (orange: upregulation, grey: no activity pattern available; white: z-score = 0, indicating upregulation of some proteins and downregulation of others), with colour intensity indicating the strength of the effect. The ratio is the proportion of proteins out of the entire pathway that were identified in the dataset, e.g. for ratio = 0.10, 10% of proteins from the pathway were identified in the dataset. LXR = liver X receptor, RXR = retinoid X receptor, FXR = Farnesoid X receptor, IL = interleukin.
