# Supplementary material for: Mastitomics, the integrated omics of bovine milk in an experimental model of Streptococcus uberis mastitis: 2. Label-free relative quantitative proteomics
Source: Mol Biosyst. 2016 Jul 14;12(9):2748–61. doi: 10.1039/c6mb00290k (PMC5048399; doi:10.1039/c6mb00290k)
Supplement: Supplementary file 1 [file MB-012-C6MB00290K-s001.zip › ESI_Figure_S1.docx]

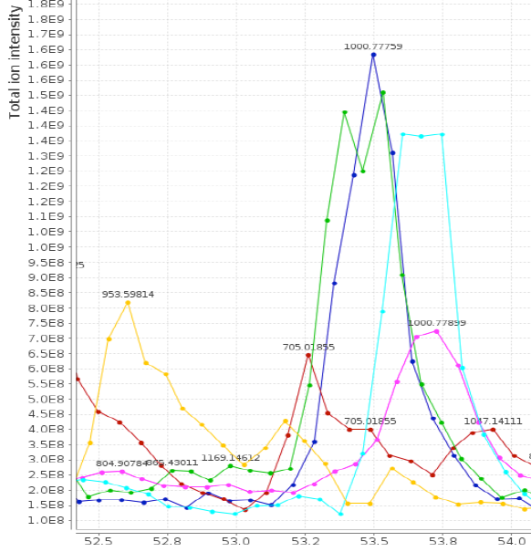

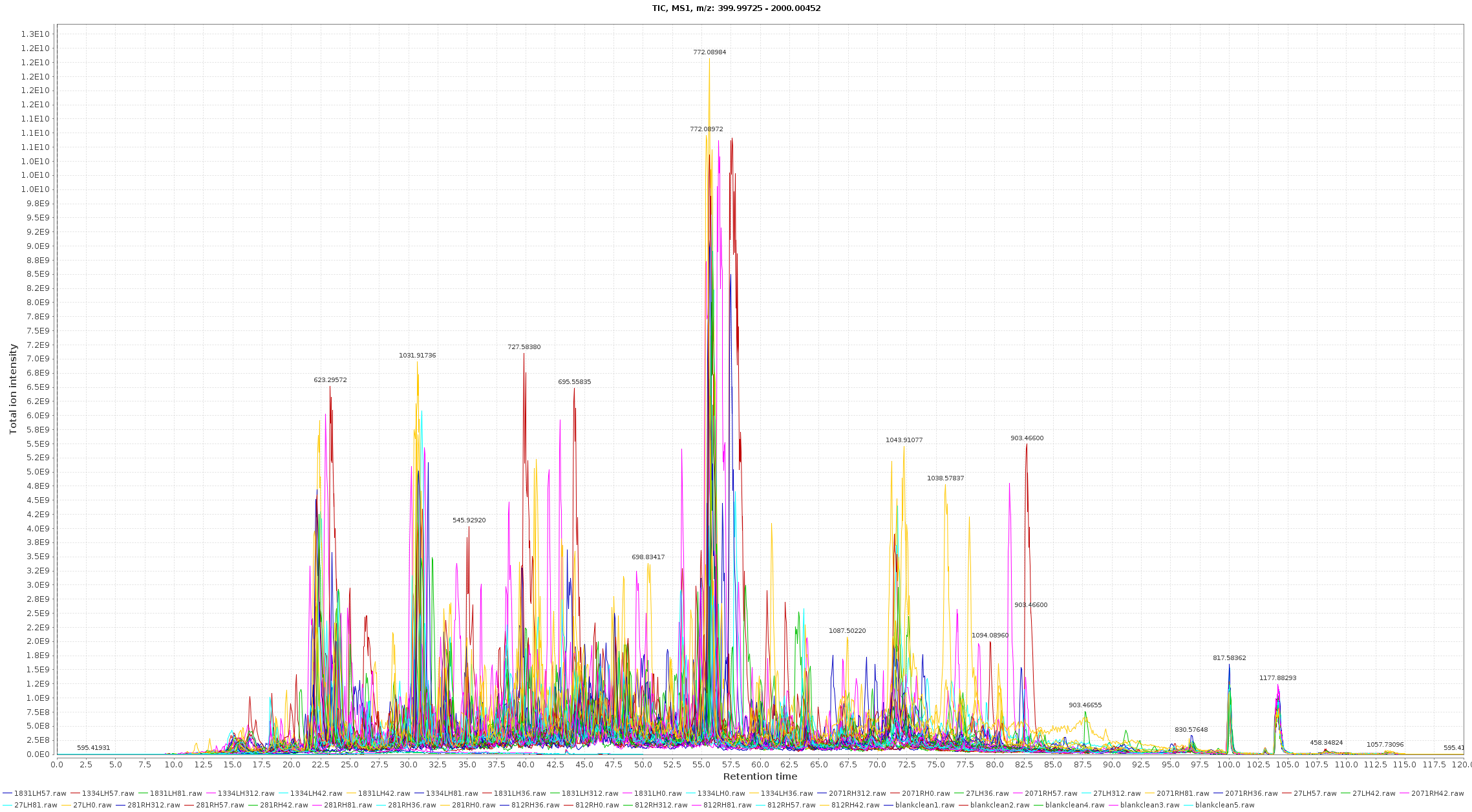


**Figure S1.** Total ion current (TIC) chromatograms for proteomic analysis of whey derived from an experimental challenge model of *Streptococcus uberis* mastitis in cattle. Colours represent individual samples, which include 6 times points from 6 cows and blanks. Inset shows a zoomed two-minute section indicating retention time drift, which was less than 2 minutes.
