# Supplementary figures and images for: Mastitomics, the integrated omics of bovine milk in an experimental model of Streptococcus uberis mastitis: 2. Label-free relative quantitative proteomics
Source: Mol Biosyst. 2016 Jul 14;12(9):2748–61. doi: 10.1039/c6mb00290k (PMC5048399; doi:10.1039/c6mb00290k)

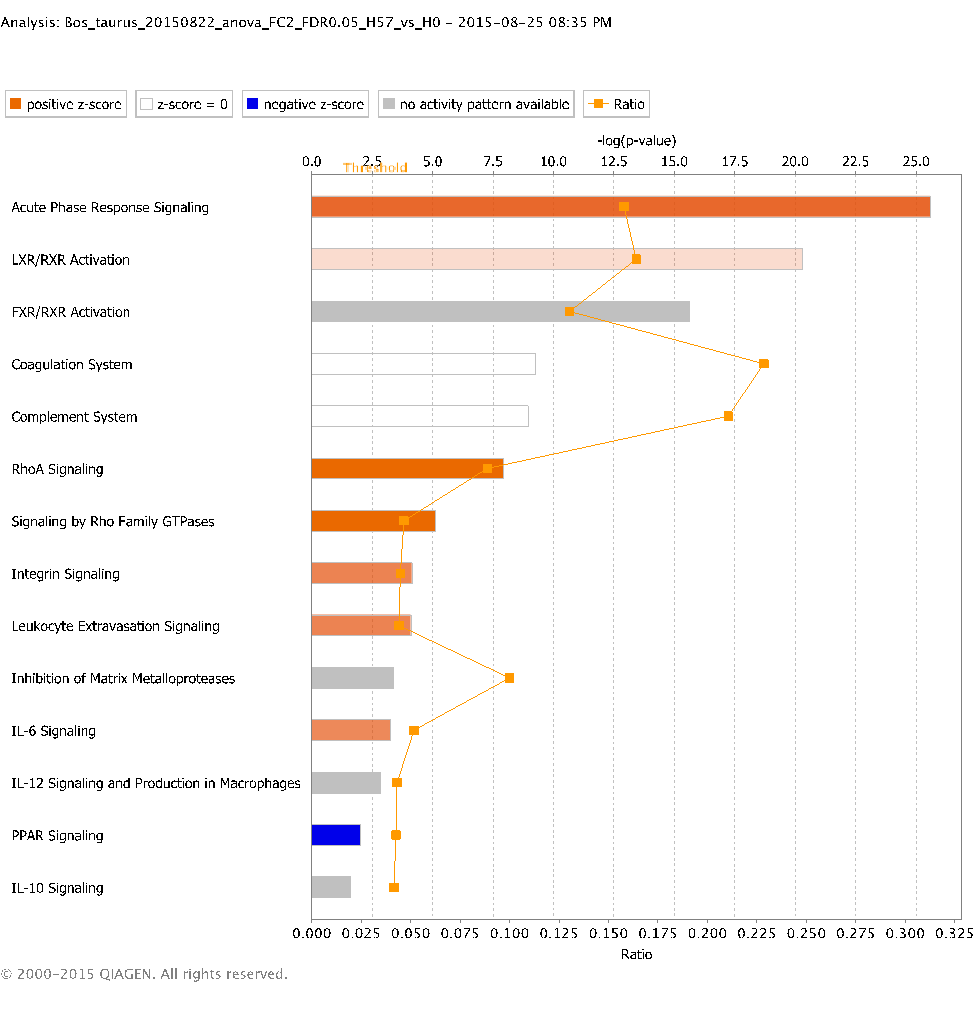

Supplement: Supplementary file 1 [file MB-012-C6MB00290K-s001.zip › ESI_Figure_S4.tif]

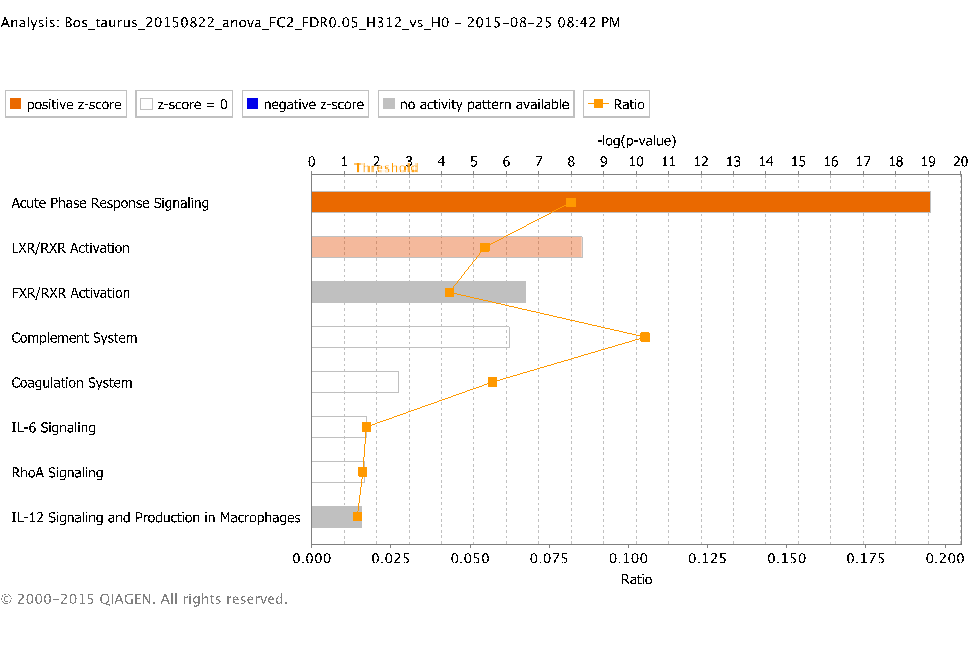

Supplement: Supplementary file 1 [file MB-012-C6MB00290K-s001.zip › ESI_Figure_S5.tif]

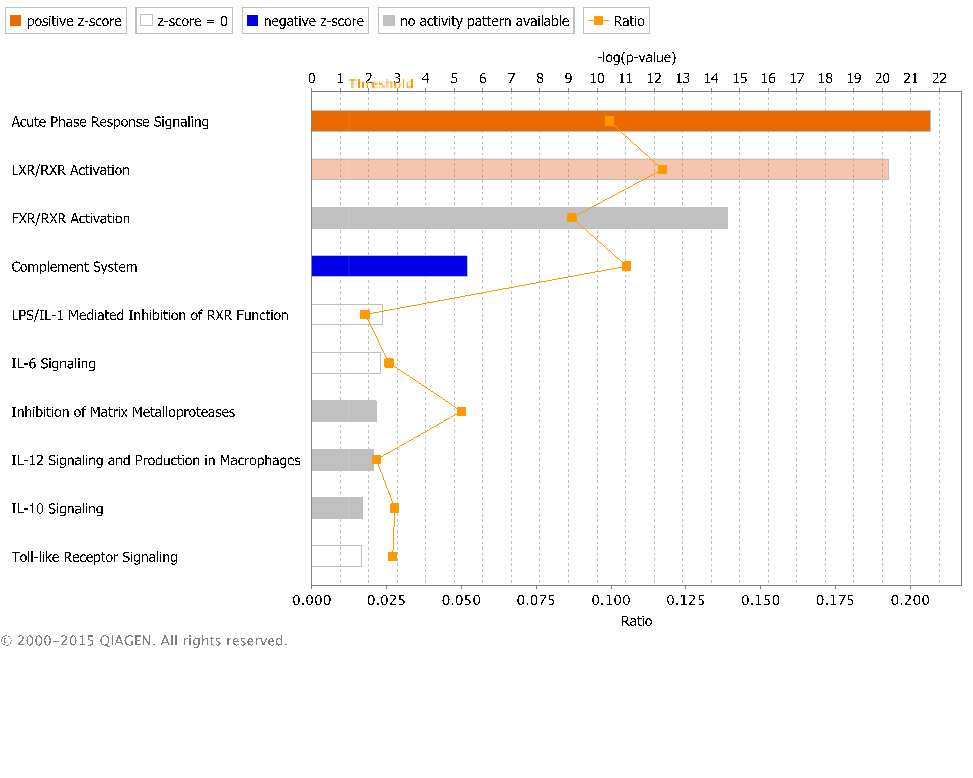

Supplement: Supplementary file 1 [file MB-012-C6MB00290K-s001.zip › ESI_Figure_S2.tif]

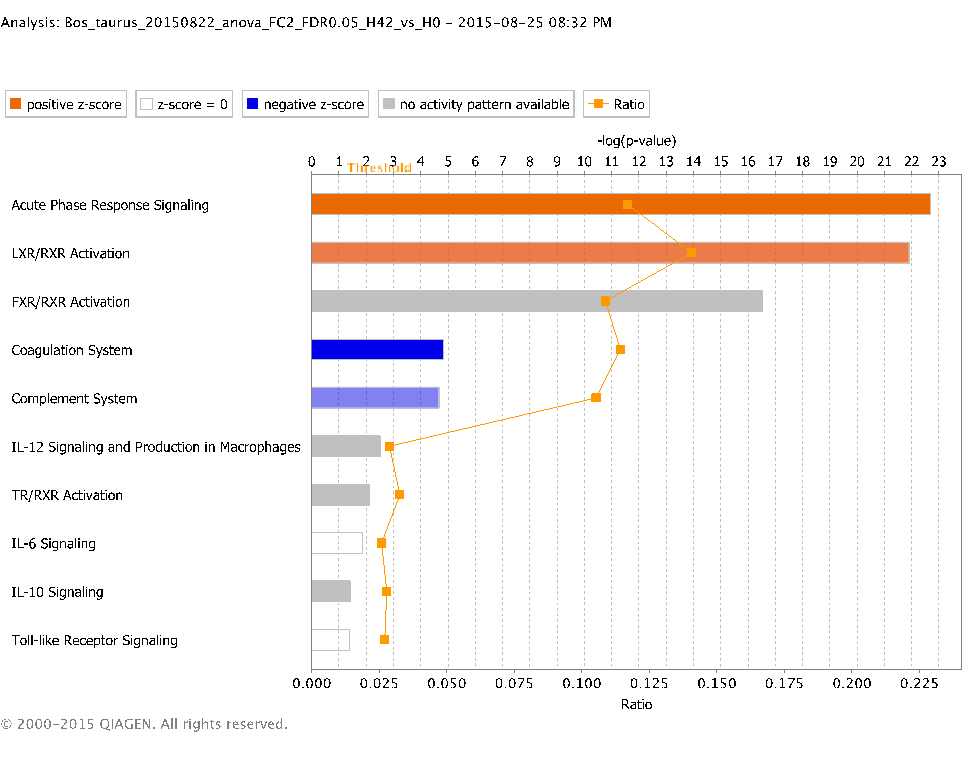

Supplement: Supplementary file 1 [file MB-012-C6MB00290K-s001.zip › ESI_Figure_S3.tif]
